# Not always positive: tactile body stimulation does not lower aggressiveness and increases stress in the Siamese fighting fish

**DOI:** 10.1007/s10695-026-01771-4

**Published:** 2026-08-17

**Authors:** Ana Carolina dos Santos Gauy, Sarah Garcia Prado, Bianca Cambiaghi e Silva, Manuel Gesto, Marta Candeias Soares, Eliane Gonçalves-de-Freitas

**Affiliations:** 1 Departamento de Ciências Biológicas, Universidade Estadual Paulista (UNESP), Rua Cristóvão Colombo 2265, São José do Rio Preto, SP 15054-000 Brazil; 2 Centro de Aquicultura da UNESP, Avenida Paulo Donato Castellane, S/N, Jaboticabal, SP 14884-900 Brazil; 3 DTU Aqua, Section for Aquaculture, Technical University of Denmark, The North Sea Science Park, Hirtshals, 9850 Denmark; 4 Marine and Environmental Sciences Centre/ARNET—Aquatic Research Network, Institute for Research and Advanced Training (IIFA), Universidade de Évora, Évora, Portugal; 5 CIBIO, Research Centre in Biodiversity and Genetic Resources – InBIO Associate Laboratory, University of Porto, Vairão Campus, Vairão, Portugal; 6 BIOPOLIS Program in Genomics, Biodiversity and Land Planning, Centro de Investigação em Biodiversidade e Recursos Genéticos, Campus de Vairão, Vairão, Portugal; 7 Associate Laboratory, School of Agriculture, CIBIO, Research Centre in Biodiversity and Genetic Resources, InBIO, University of Lisbon (ISA), Lisbon, 1349-017 Portugal

**Keywords:** Sensory enrichment, Ornamental fish, Welfare, Cortisol, Dopamine, Serotonin

## Abstract

Artificial body tactile stimulation (TS) has been shown to improve fish welfare by reducing aggressive behavior and stress, thereby serving as a sensory enrichment. Here, we tested the effect of TS on aggressive behavior and stress response in the Siamese fighting fish (*Betta splendens*), an ornamental species reared worldwide and renowned for its high aggressiveness. Twenty isolated male fish were exposed either to TS (provided by a frame with vertical plastic sticks lined with silicone bristles, inserted in the middle of the tank) or to a control treatment (sticks without bristles) for 22 days. Individual aggressive behavior was evaluated before and after the treatment period using mirror tests and staged pair fights. We measured cortisol, brain serotonin (5-HT), and dopamine (DA) to infer stress and possible associations with aggressiveness and the reward system. Although fish actively interacted with the bristles, TS did not alter overt physical fighting but significantly increased frontal and lateral displays. Furthermore, TS elevated cortisol levels, decreased 5-HT, and increased the 5-HIAA/5-HT turnover ratio. DA levels remained unchanged, indicating no activation of neural reward pathways. Contrary to our expectations, TS did not mitigate aggressiveness in this species but rather seemed to prompt an increase in both behavioral and physiological reactivity (cortisol levels and 5-HT activity) to fighting. Our findings indicate that artificial selection for high aggression in *B. splendens* may alter sensory processing to touch, causing physical contact to act as a stressor rather than an enrichment.

**Supplementary Information:**

The online version contains supplementary material available at https://doi.org/10.1007/s10695-026-01771-4.

## Introduction

Aquaculture of ornamental fish is among the fastest-growing activities, with more than 1.5 billion fish used *per year* worldwide (FAO 2024). The trade activity of ornamental fish, however, presents several conditions that affect fish welfare. Some of the main problems include stress induced by capture, transport, and farming practices, mainly in high densities and low-quality waters, which can cause chronic stress, damage to the immune system, reduced reproductive capacity, and affect the fish’s health (Huntingford et al. 2006; Maia et al. 2025; Planellas et al. 2026). The stressful conditions vary between species, trade phases, and the environments in which fish are kept (Torgersen 2020). Therefore, finding strategies to reduce negative impacts on the quality of life is critical.

One way to mitigate the effects of the artificial environment on fish welfare is by using tools and structures that promote different types of environmental enrichment, and enhance the complexity of the environment, namely by adding physical, sensory, occupational, social, dietary, and cognitive enrichment (Näslund & Johnsson 2016; Arechavala-Lopez et al. 2022; Kleiber et al. 2023). Environmental enrichment for fish involves modifying their captive environment to meet their behavioral, physiological, and psychological needs, aiming to reduce stress, stimulate natural behaviors, and increase the likelihood that fish experience positive affective states (Brydges and Braithwaite 2009; Arechavala-Lopez et al. 2022). In this context, one type of sensory enrichment that has gained attention in recent years is the application of tactile stimulation (TS), similar to therapeutic massage in humans or gentle touch in vertebrate social interactions. This strategy uses artificial or natural mechanical stimuli to parts of the animal’s body, aiming to promote positive effects on the receiving individual (Soares et al. 2011). TS, when performed on humans, has therapeutic properties, causing stress reduction and immune system strengthening (Charnetski et al. 2004; Field 1995). This stimulation also affects other vertebrate animals, such as cows and bulls (Schmied et al. 2010; Probst et al. 2013), piglets (Tallet et al. 2014), and dogs (Rehn et al. 2014). TS is also complementary to the treatment of depressive disorders, as it helps increase serotonin levels, a neurotransmitter that is essential for regulating mood and overall animal welfare (Field et al. 2005).

It is known that artificial TS reduces stress in a tropical reef species (Soares et al. 2011) and decreases fear in zebrafish (Schirmer et al. 2013)*.* In the cichlid fish Nile tilapia, TS reduced aggression in previously isolated individuals (Bolognesi et al. 2019) as well as in social groups (Gauy et al. 2022). It also increases growth rate and feeding efficiency (Gauy et al. 2022) and counteracts the effects of high stocking densities (Gauy et al. 2023). Furthermore, TS provided by air bubble curtains is associated with increased welfare in rainbow trout fry and fingerlings by reducing aggressive and abnormal behaviors, improving learning performance, and attenuating emotional-like fear reactions (Amichaud et al. 2024). Recently, Soares et al. (2026) found a reduced stress response in gilthead seabream (*Sparus aurata*) after TS enrichment. These positive effects of TS highlight the relevance of such sensory enrichment as a tool for improving the welfare of ornamental fish. However, it is crucial to test the effectiveness of TS in different species, as the effects of the same enrichment can vary depending on the species’ natural behavior, stimulus perception, and the way the enrichment is presented (Arechavala-Lopez et al. 2022; Winberg and Sneddon 2022).

The calming effect of TS in mammals has been attributed to skin mechanoreceptors and afferent C-tactile (CT) fibers that, when fired, release neurotransmitters such as dopamine (DA), serotonin (5-HT), oxytocin (OT) (Walker and McGlone 2013; Reinig et al., 2017) and endocannabinoids (Jones et al. 2025) in different brain areas. These CT fibers are associated with gentle and pleasant touch instead of discriminative touch (Schirmer et al. 2023). In fish, the CT fibers are not identified, although mechanosensory cells are distributed along the lateral line, fins, and other regions of the skin (Butler and Maruska 2016; Devitsina and Lapshin 2020). Nevertheless, similar effects have already been described. For example, mechano-stimulation in fish is also associated with endocannabinoids, suggesting a hedonic effect of TS (Maximino et al. 2025). Moreover, Abreu et al. (2018) found that in mutualistic cleaner/client interaction, fish exposed to a cleaner showed higher levels of DA in the forebrain and higher levels of its main oxidative metabolite, DOPAC, in the brainstem, suggesting a rewarding effect of TS provided by cleaner fish during such interaction. Another important monoamine associated with cooperative and social behavior is 5-HT (Paula et al. 2015; Abreu et al. 2018), which reduces aggressive interactions in fish (Le Page et al. 2005; Winberg and Thörnqvist 2016). Nevertheless, its effect on behavior following the TS is unknown. However, as the neural pathways of mechanical sensation and their connections with the hypothalamus-pituitary axis are well conserved across vertebrates, it is plausible to assume that the effects of TS observed in mammals would also be observed in teleost fishes.

Building on the positive effects of TS in several species, we tested its possible effect on the aggressive behavior of the Siamese fighting fish, *Betta splendens* Regan, 1823, one of the most popular and commercially important ornamental species worldwide (Thongprajukaew et al. 2011; Oliveira et al. 2022), also widely used in experimental research (Lichak et al. 2022). This species has been constantly subjected to artificial selection in captive environments (Smith 1945; Iwata et al. 2021), resulting in a diversity of body colorations and fin sizes that today present a striking contrast compared to wild species, which have more discreet colors and smaller fins (Zhang et al., 2022). Long-term selective breeding among different lineages for vibrant body color also selected highly aggressive individuals (Ramos & Gonçalves 2019). Furthermore, there are records that aggressive individuals have been breeding for entertainment and used in clandestine fights for more than 600 years (Sermwatanakul 2019). Such intense selection has produced highly aggressive phenotypes compared to wild types (Verbeek et al. 2007).

When facing an opponent, *B. splendens* exhibits aggressive behavior, which usually starts with threat displays like operculum opening and lateral tail-beating movements, and then escalates to overt fights that include bites and tail beating (Simpson et al., 1968). These aggressive interactions between conspecific males are adaptive behaviors, crucial to establish hierarchies and influence access to environmental resources, such as food and sexual partners (Gonçalves-de-Freitas et al. 2019; Winberg and Sneddon 2022). However, in artificially selected bettas, the aggressiveness became nonadaptive, so that fish are usually kept isolated in small glass tanks (Clark-Shen et al. 2024); otherwise, males can fight each other until death. Physical enrichment was shown to reduce juveniles and subadult bettas’ aggressive behavior, but was not efficient in adults (Iwata et al. 2021), which show increased aggression instead. However, the effect of TS has not been tested in this species yet. Here, we hypothesized that access to TS impacts the aggressive behavior of *B. splendens*, changes social stress levels, and activates monoaminergic pathways. If aggression is reduced, we would expect to observe reduced stress and 5-HT levels and a putative increase of DA and its metabolites by activating the fish reward system. Therefore, we tested the effects of TS on aggressiveness and stress response, as well as on brain DA and 5-HT in *B. splendens*.

## Methods

### Housing conditions

Adult male *Betta splendens* Regan 1823 (Anabantiformes, Osphronemidae) were purchased from a local commercial supplier, where each fish was raised isolated from its conspecifics since the juvenile stage. In the laboratory, the specimens were kept isolated for 15 days in polycarbonate tanks (10 L), in a Zebrafish Rack Altamar, for acclimatization and control of possible pathogens from the breeding site. The Zebrafish Rack provided a complete filtration system, including a self-cleaning mechanical filter, UV-C disinfection, biological filter, activated carbon filter, and automatic water change to ensure the maintenance of water quality. The water temperature was controlled to 28 °C, and the photoperiod to 12:12 h (lights on from 7:00 a.m. to 7:00 p.m.). The animals were fed twice a day (9:00 a.m. and 3:00 p.m.) with floating food specific to bettas, following the quantities recommended by the supplier (4 grains of food per feeding period), corresponding to 1.6% of the biomass/day.

### Tactile stimulation apparatus

**Fig. 1:**
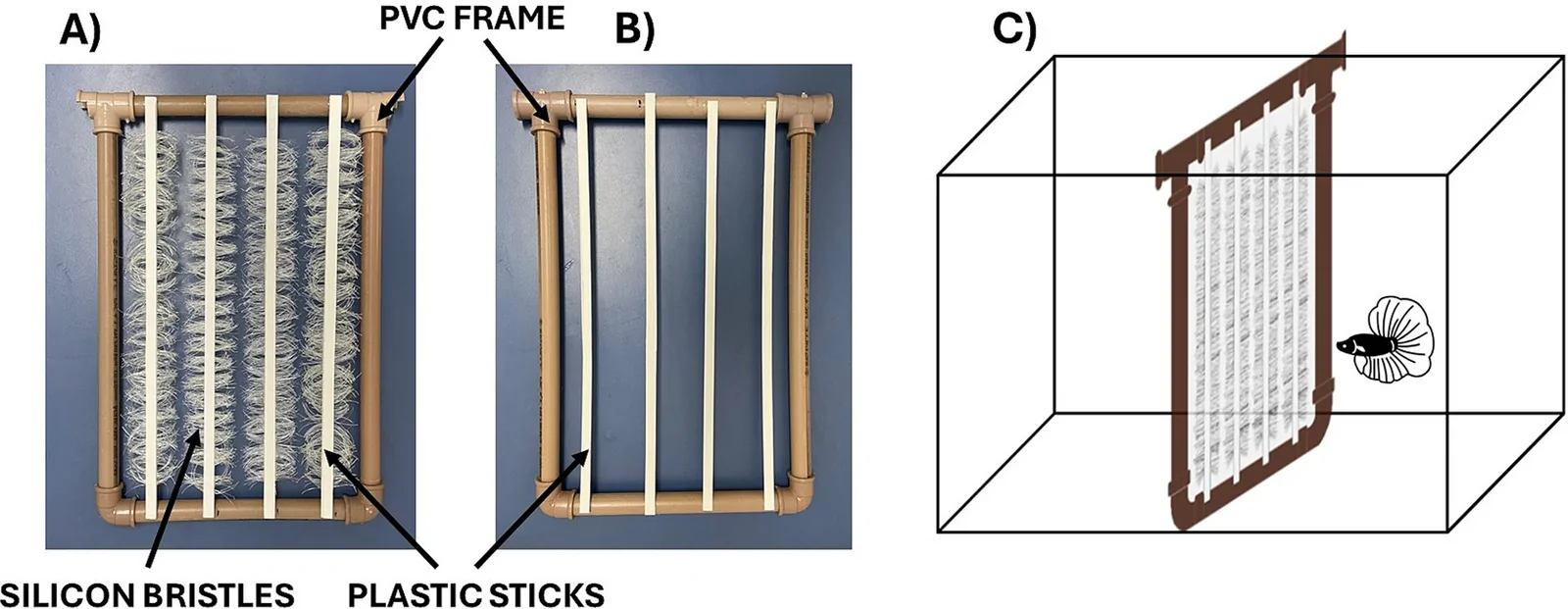
Tactile stimulation device. The TS apparatus consisted of a rectangular PVC frame filled with vertical plastic sticks, with silicone bristles on their sides that promote TS (**A**) and without silicone bristles for control (**B**). Both apparatuses were inserted in the middle of the tank, and fish received TS when passing through the bristles (**C**)

TS was done by an apparatus previously validated in our lab for Nile tilapia (Bolognesi et al. 2019; Gauy et al. 2022), made of a rectangular PVC structure with vertical plastic sticks, filled with silicone bristles on their sides (Fig. 1A). A similar device, but without silicone bristles, was used as a control (Fig. 1B). Both devices were inserted in the center of the aquarium (Fig. 1C), and physical stimulation was achieved only when a fish passed between the bristles. The silicone bristles were chosen due to their softness, which does not cause bodily harm or remove the mucus that protects the fish’s body (Bolognesi et al. 2019).

### Experimental design

**Fig. 2:**
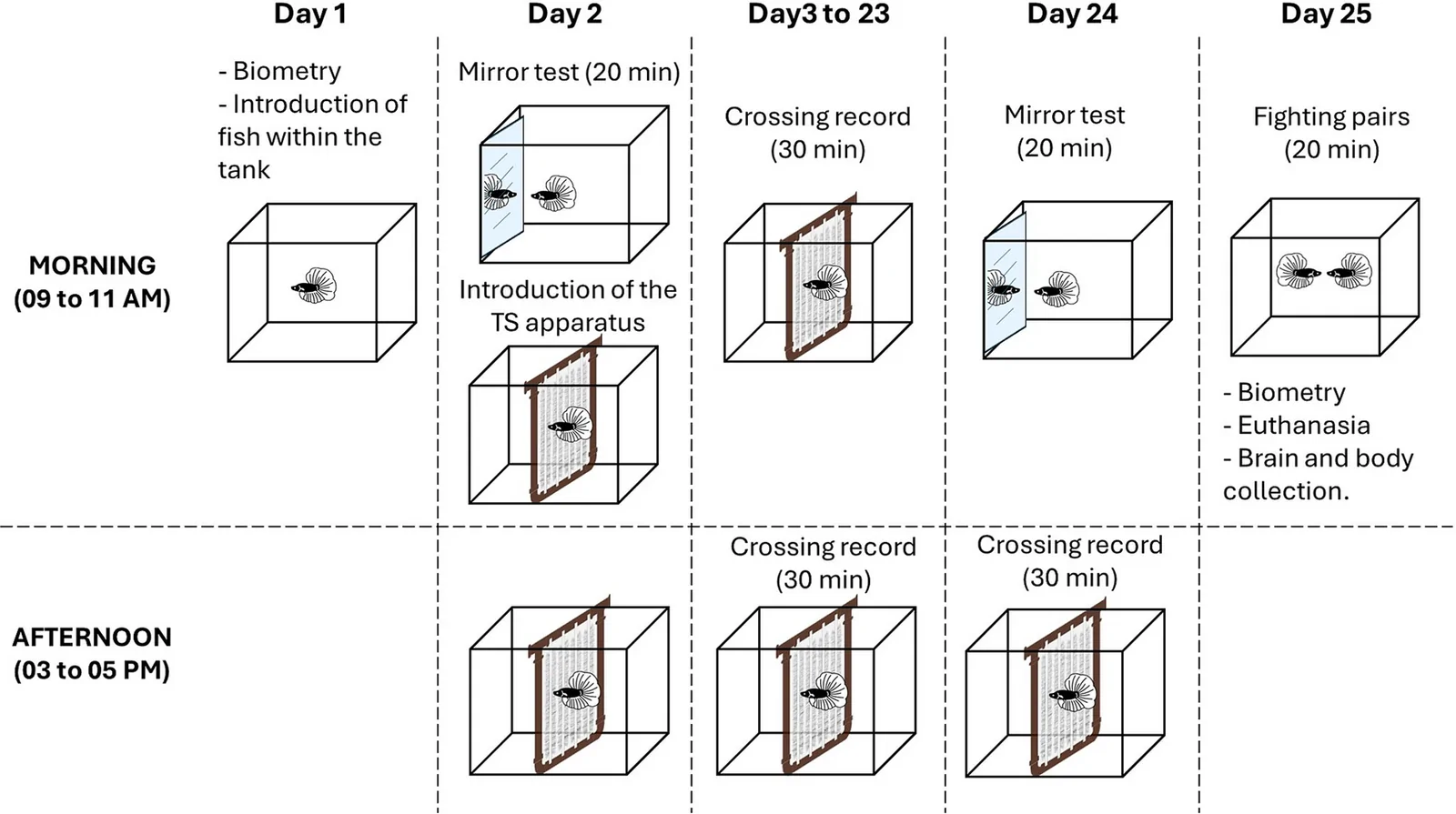
Timeline for experimental protocol in *Betta splendens.* On Day 1, fish were isolated in glass tanks to adjust to the new environment. On Day 2, baseline aggressive motivation was assessed using a mirror test. Fish then underwent 22 days of either tactile stimulation (TS) or no stimulation (control), during which they were video recorded twice a day to quantify crossings through the apparatus. Following this period, fish were retested in the mirror, after which the TS apparatus was reintroduced. On the following day, fish within the same treatment group were paired in dyadic fights to evaluate aggressive behavior. On the final day, individuals were euthanized via deep anesthesia, and their brains and bodies were frozen (− 80 °C) for subsequent assays. The layout illustrates the treatment with TS, but the control group followed an identical protocol with an apparatus without silicone bristles

Isolated adult fish were assigned to either a treatment with TS or a control without TS for 22 days (*N* = 20 replicates each). Aggressive behavior was tested both individually by the mirror test, before and after TS (or control), and in pairs’ contests at the end of the TS period. Although fights with a real opponent and with a mirror image are similar in adult *B. splendens* (Arnott et al. 2016), we used both methods to evaluate aggressiveness, particularly the mirror, to evaluate before and after TS. After the last aggressive behavior test, fish brains and bodies were frozen to later assay monoamines and cortisol, respectively. A summary of the protocol is in Fig. 2.

Fish were tested in glass tanks (30 × 40 × 30 cm; ~ 36 L) whose sides and back were covered with blue plastic to prevent visual contact among fish in neighboring tanks. The choice of blue color was based on studies that indicate an apparent decrease in cortisol levels in other fish species (Maia & Volpato 2013). Only the front was free for observation and video recording. The water level was maintained at 20 cm, like in the natural environment (Jaroensutasinee and Jaroensutasinee 2001). We did not use a biological filter or aerator during the experimental period, as the Betta fish perform aerial respiration (Goldstein 2015; Tate et al. 2017). Nevertheless, water quality parameters were controlled: temperature maintained at 28 °C; pH at 7.5; and ammonia and nitrite near to 0.0 ppm (measured by commercial kits, Labcon Test).

On the first day, the fish were anesthetized with benzocaine (0.18 g/L), measured, weighed, photographed for further identification, and randomly assigned to one of the treatments. The initial length and weight mean values of the animals were, respectively, for TS (mean ± SE: 3.44 ± 0.05 cm; 1.31 ± 0.08 g) and the control (mean ± SE: 3.42 ± 0.05 cm; 1.23 ± 0.05 g).

On the second day, a mirror was introduced in the tank, and the fish were video recorded (20 min) to assess the previous level of individual aggression. Then, the mirror was removed, and the apparatuses (with or without TS) were introduced in the tank, wherein fish were kept for the next 22 days. The following day, fish were tested for individual aggression again with a second mirror test (20-min recording). During the mirror tests, the device was removed to avoid any interference and was replaced after the test.

On the 25th day, two fish from the same treatment, of similar size and weight, were placed together in a neutral aquarium and filmed for 20 min to evaluate the fighting against a real opponent (dyadic fights). Video recording of aggressive interactions with the mirror and between pairs was carried out with cameras placed in front of the tank. After the dyadic fight, the fish were anesthetized with the previously mentioned dose and underwent biometry again. They were euthanized by deepening anesthesia (benzocaine 0.36 g/L). We then collected the heads and bodies of the individuals and stored them at − 80° C for later cortisol and brain monoamines assays.

### Efficiency of use of the apparatus

The efficiency of the apparatus was assessed by quantifying the number of times fish from both treatments crossed the apparatus over 22 days. During this time, individuals were filmed daily in the morning (09:00–11:00 a.m.) and in the afternoon (03:00–05:00 p.m.), 30 min each period. Morning and afternoon recordings were used because we did not know whether the daily rhythm influenced swimming behavior. Nevertheless, we analyzed the sum of the two periods to consider a better sample of crossings. A video camera was positioned above each aquarium, allowing remote observation of the fish and the capture of videos of daily crossings. The apparatus was considered efficient if fish passed through the bristles constantly over the days.

### Aggressive interaction

Aggressive interactions were based on ethograms previously described for *Betta splendens* in Simpson (1968), Ramos and Gonçalves (2019), and Silva et al. (2025), with some adjustments regarding fights against the mirror image and a real opponent (Table 1). These aggressive interactions were categorized into attacks (overt fights) and threat displays (restrained fights) due to the greater energetic expenditure in attacks than in threats (Haller and Wittenberger 1988; Castro et al. 2006) and because restrained fights can escalate to overt ones during contests (Arnott et al. 2016). Attacks involve direct physical contact, whereas threat displays are aggressive units that do not involve direct physical contact (Ros et al. 2006). Some behaviors, such as mouth fighting, charge, and flight, are not observed in the mirror test due to the impossibility of performing these behaviors against a virtual opponent (Table 1).

**Table 1:** Ethogram of the aggressive behavior of *Betta splendens*, both with a mirror image and with a real opponent. Based on and modified from Simpson (1968), Ramos and Gonçalves (2019), and Silva et al. (2025)

Category	Aggressive unity	Interaction with a mirror image	Interaction with a real opponent
Threat displays (restrained fights)	Frontal display	An individual faces its image with unpaired fins and/or opercula extended	An individual faces the opponent with unpaired fins and/or opercula extended
	Lateral display	Extension of fins and opercula when laterally positioned near the mirror	Extension of fins and opercula when laterally positioned near the opponent, without touching it
	Body undulation	Waves of the body and caudal fin near the mirror, without touching it	Waves of the body and caudal fins without touching the opponent fish
	Charge	---	Fast approach toward the opponent, without touching it
Attacks (overt fights)	Bites (mouth hits)	Swim towards the mirror and bite it with its mouth	Swim towards the opponent and bite its body with the mouth
	Mouth fight (jaw lock)	---	Individuals join their mouths and push each other with their mouths locked. They can stay in the middle water column or go to the bottom
	Tail beating	Waves the caudal fin towards the mirror	Waves the caudal fin towards the opponent side, touching its body
Avoidance behavior	Flight	---	An individual swims quickly away from the opponent, avoiding being attacked

### Brain monoamines and cortisol assay

After euthanasia by deepening anesthesia, we separated the heads from the bodies of the fish from both treatments and froze them at − 80 °C. Heads were removed from the freezer and whole brains were dissected out while still semi-frozen. Serotonin (5-hydroxytryptamine, 5-HT) and DA, as well as their main metabolites, 5-HIAA and DOPAC, respectively, were assayed. The assay was performed by HPLC-EC (high-performance liquid chromatography with electrochemical detection) according to Luiz et al. (2026). Cortisol was extracted from fish whole bodies (without the head and without all fins but pectorals) by means of liquid–liquid extraction using ethyl acetate (CAS 141–78-6). Bodies were finely minced while still frozen on an ice-cold petri dish. Minced tissue was further homogenized by ultrasonication (Sonopuls HD3100, Bandelin Electronic, Germany) in two volumes of phosphate-buffered saline (PBS) 150 mM, pH 7.4. A volume of ethyl acetate (double than the PBS volume) was added to the homogenate, and the mixture was vortexed vigorously for 1 min. The homogenate was then centrifuged (2000 × *g*, 10 min, room temperature) and 1 mL of the organic phase (upper phase) was collected into a clean tube and evaporated in a vacuum centrifuge (60 min at 50 °C). Extracts were reconstituted in 200 µL of ELISA extraction buffer (Neogen) and stored at − 80 °C until analysis by ELISA (cortisol ELISA kit, ref: 402,710, Neogen Europe, Ayrshire, UK).

### Growth parameters

The following parameters related to the growth of each individual were examined: (1) length gain (final-initial standard length) and (2) weight gain (final-initial body weight). In addition, we evaluated the specific growth rate (SGR) = [(ln Final weight − ln Initial weight/Time)*100] and Fulton’s Condition Factor (K ) = (weight/standard length^3^).100.

### Data analysis

Data were assessed for normality using the Kolmogorov–Smirnov test and homoscedasticity using Levene test (Zar 2010). When necessary, data were square root transformed to achieve homoscedasticity. Variables were analyzed as follows:

- The frequency of crossings through the apparatus was analyzed by mixed model ANOVA, with treatments as categorical variables and observations over time as repeated measures.
- Aggressive interaction with the mirror was also analyzed by mixed model ANOVA, with treatments as categorical factors and the periods before and after TS (or control), as repeated measures. Variables analyzed include latency to start threats and attacks as well as the frequency of threat displays, attacks, and total fights (attacks + threats). Tukey post hoc test was applied for multiple comparisons.
- Dyadic fights were analyzed according to the social rank (winner and loser) in each treatment by two-way ANOVA, considering the rank and treatments with or without TS as categorical factors and behavior and physiological variables as dependent ones. The same was done to monoamines and cortisol levels, which were quantified in 10 replicates per treatment due to logistical reasons. Tukey post hoc test was applied for multiple comparisons when necessary.
- We compared the latency to start displays and to start attacks in dyadic fights, as well as growth between the treatments using unpaired *t*-test.
- Condition factor (K) was also analyzed by mixed model ANOVA and Tukey post hoc test.
- We tested Spearman rank correlation between aggressive behavior and physiological variables (cortisol, DA, 5-HT, and their metabolites). *P*-values from the correlation analyses were adjusted for multiple comparisons using the Holm-Bonferroni sequential procedure (Holm 1979). The Holm-Bonferroni procedure was preferred over the classical Bonferroni correction because it controls the family-wise error rate while being less conservative, thereby reducing the risk of type II errors (Holm 1979; Aickin & Gensler 1996). To avoid redundancy, we tested only threat displays, attacks, latency to displays, and latency to attacks, so that each physiological variable was tested 4 times. Statistical significance was set at *p* ≤ 0.05.

### Ethical note

This study was developed according to the Ethical Principles in Animal Experimentation established by the National Council for the Control of Animal Experimentation (CONCEA) and received approval from the Committee of Ethics on the Use of Animals of the Institute of Biosciences, Letters and Exact Sciences of UNESP, São José do Rio Preto, SP (CEUA IBILCE/UNESP), permit # 246/2023.

## Results

### Crossings through the TS apparatus

**Fig. 3:**
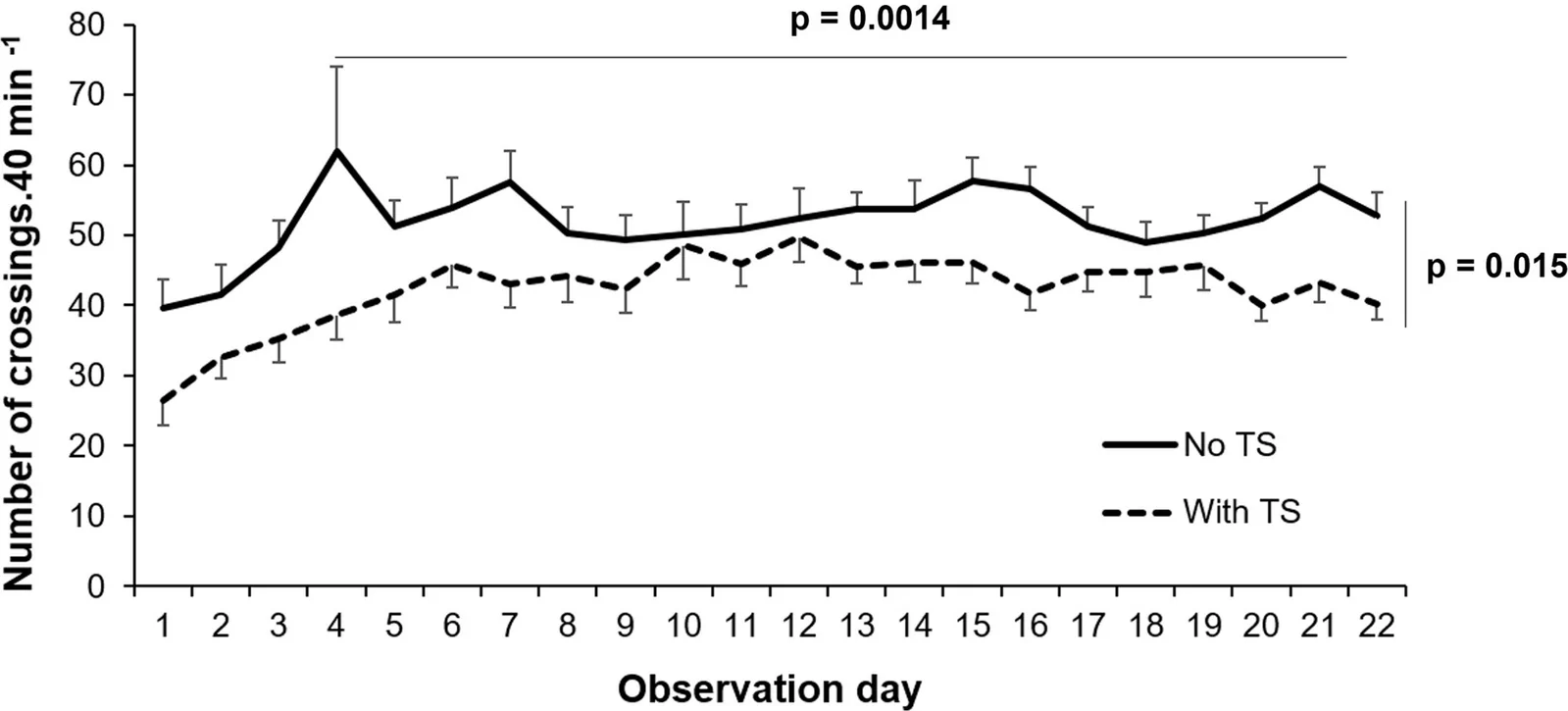
Apparatus efficiency. Number of crossings by the apparatus with or without silicone bristles over the 22 days (*N* = 20 individuals by treatment), summed recordings in morning and afternoon. Data are mean ± SE compared by ANOVA for repeated measures, followed by Tukey post hoc test. *P*-value indicates a significant difference from the three previous days (upper line) and between treatments (lateral line)

Fish crossed the apparatus slightly more frequently in the control group than in the TS one (ANOVA for repeated measures, F_(1,38)_ = 6.44; *p* = 0.015). There were significant differences in crossings over time (F_(1,21)_ = 5.11; *p* = 0.000001), which increased until the 3rd day, and remained stable afterwards (*p* = 0.0014) (Fig. 3). There was no statistical interaction between treatments and periods (F _(1,21)_ = 1.53, *p* = 0.06).

### Aggressive behavior

#### Mirror test

No significant differences were observed for all aggressive units recorded. Therefore, we showed here those behaviors grouped in attacks and displays, and the latency to start attacks and displays, before and after the introduction of the apparatuses inside the tanks (Table 2).

**Table 2:** Number of aggressive behaviors (in 20 min) in contests of *B. splendens* with a mirror before and 22 days after the apparatus introduction, both with and without tactile stimulation (TS). Data are mean ± SE and were analyzed by ANOVA for repeated measures (Mixed Model), considering the treatments with and without TS as categorical factors, and before and after apparatus introduction as repeated measures. *N* = 20 individuals by treatment

	Treatments					
	Without TS		With TS		ANOVA	
Aggressive variables	Before	After	Before	After	F_***(1,38)***_	*P****-***value
Displays	104.45 ± 7.98	107.30 ± 10.06	96.75 ± 8.63	105.65 ± 12.34	Between treatments = 0.34	0.56
					Within treatments = 0.13	0.72
					Interaction = 0.08	0.78
Attacks	62.40 ± 11.64	60.90 ± 18.79	39.95 ± 8.58	31.70 ± 11.16	Between treatments = 1.62	0.21
					Within treatments = 3.19	0.08
					Interaction = 0.02	0.88
Total confrontation	166.85 ± 14.93	168.20 ± 22.58	136.70 ± 2.31	137.35 ± 17.60	Between treatments = 2.5	0.12
					Within treatments = 0.09	0.76
					Interaction = 0.01	0.91
Latency to displays	18.40 ± 3.10	15.55 ± 3.01	27.50 ± 7.46	20.55 ± 8.53	Between treatments = 0.48	0.49
					Within treatments = 1.43	0.24
					Interaction = 0.20	0.66
Latency to attacks	417.10 ± 94.91	579.00 ± 103.00	568.00 ± 8.86	698.35 ± 95.80	Between treatments = 2.44	0.13
					Within treatments = 3.34	0.08
					Interaction = 0.08	0.78

#### Fight in pairs

We compared aggressive variables between winner and loser fish and treatments in dyadic fights. Only the frontal and lateral displays were higher in fish that experienced TS (Table 3). No other behavioral unit showed significant differences between treatments, although some effect of rank was found (Table 3). As expected, most of the aggressive behavior was higher in winners than in losers, except for flight, which was reduced in winners. Caudal undulation, mouth fight, tail beats, and bites showed no differences at all. Furthermore, no significance was found between treatments either for the latency to start displays (TS = 48.40 ± 10.56 s; without TS = 25.30 ± 4.03 s; *t* = − 1.85; *p* = 0.08) nor to start attacks (TS = 392.30 ± 105.43 s; without TS = 466 ± 77.84; *t* = − 0.87; *p* = 0.40).

**Table 3:** Number of aggressive behavior units (in 20 min) in winners and losers of *B. splendens* after dyadic contests in treatments with or without tactile stimulation (TS). Data are mean ± SE. two-way ANOVA. N = 10 pairs for each treatment (5 winners and 5 losers)

	Treatments					
	Without TS		With TS		two-way ANOVA	
Aggressive variables	Winners	Losers	Winners	Losers	F_***(1,36)***_	*P****-***value
Frontal display	15.30 ± 3.88	4.60 ± 0.93	26.40 ± 6.30	8.30 ± 1.73	Between treatments = 4.17	**0.048**
					Between ranks = 17.2	**0.002**
					Interaction = 0.29	0.59
Lateral display	26.70 ± 2.50	20.00 ± 3.40	37.40 ± 3.23	34.40 ± 6.07	Between treatments = 6.33	**0.02**
					Between ranks = 2.55	0.11
					Interaction = 0.12	0.73
Caudal undulation	22.50 ± 5.55	18.20 ± 5.00	18.30 ± 4.29	15.40 ± 4.34	Between treatments = 0.24	0.62
					Between ranks = 0.61	0.44
					Interaction = 0.004	0.95
Charge	12.80 ± 5.31	1.00 ± 0.33	5.80 ± 3.46	0.80 ± 0.47	Between treatments = 2.10	0.12
					Between ranks = 9.73	**0.005**
					Interaction = 1.30	0.93
Bites	15.90 ± 4.13	13.30 ± 3.67	12.20 ± 3.09	11.00 ± 3.54	Between treatments = 0.60	0.44
					Between ranks = 0.53	0.47
					Interaction = 0.01	0.91
Mouth fight	1.10 ± 0.77	1.00 ± 0.68	0.80 ± 0.42	0.70 ± 0.40	Between treatments = 0.01	0.92
					Between ranks = 0.02	0.88
					Interaction = 0.002	0.97
Tail beat	12.20 ± 3.30	9.10 ± 3.27	9.90 ± 2.66	7.40 ± 2.55	Between treatments = 0.21	0.65
					Between ranks = 0.98	0.33
					Interaction = 0.006	0.94
Flight	1.10 ± 0.53	18.40 ± 7.16	0.80 ± 0.47	7.70 ± 3.47	Between treatments = 1.89	0.18
					Between ranks = 10.97	**0.002**
					Interaction = 1.36	0.25
Displays	64.50 ± 4.50	42.80 ± 7,42	82.10 ± 7.44	58.10 ± 10.51	Between treatments = 2.49	0.12
					Between ranks = 8.79	**0.005**
					Interaction = 0.008	0.93
Attacks	42.00 ± 7.63	24.40 ± 6,35	28.70 ± 4.19	19.90 ± 5.66	Between treatments = 1.22	0.28
					Between ranks = 5.61	**0.023**
					Interaction = 0.15	0.70
Total confrontation	106.50 ± 10.49	67.20 ± 12.63	110.80 ± 9.00	78.00 ± 14.37	Between treatments = 0.29	0.59
					Between ranks = 9.12	**0.0046**
					Interaction = 0.04	0.84

### Brain monoamines

**Fig. 4:**
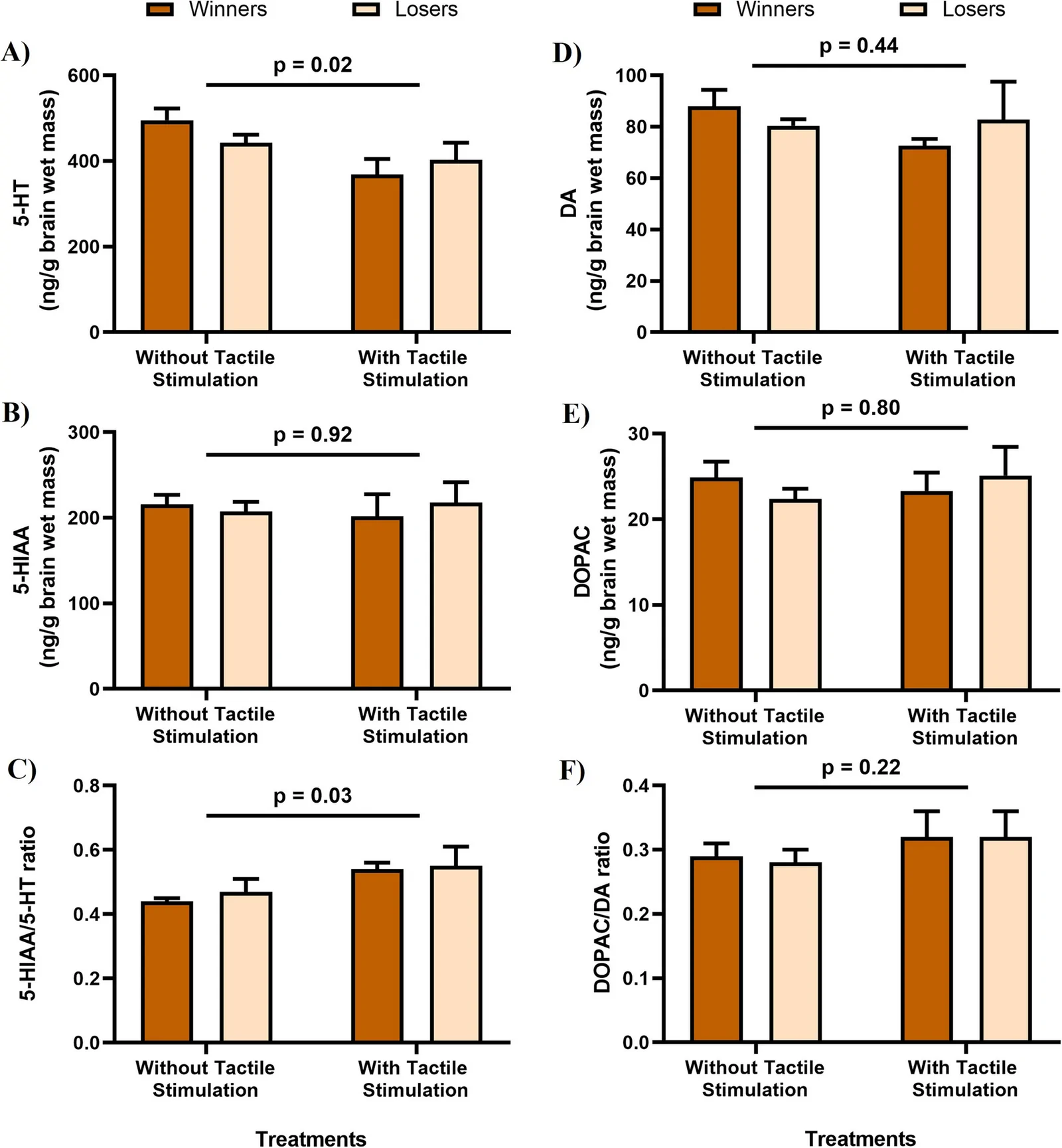
Concentration of whole brain monoamines and their metabolites. **A** Serotonin (5-HT); **B** 5-hydroxyindoleacetic acid (5HIAA); **C** ratio of 5HIAA/5-HT; **D** dopamine (DA); **E** 3,4-dihydroxyphenylacetic acid (DOPAC); **F** DOPAC/DA ratio. Values are shown for winner and loser fish. *P*-value between bars compares between treatments after two-way ANOVA (*N* = 10 pairs, being 5 winners and 5 losers *per* treatment). There was no effect of rank. Data are mean ± S.E

Only two significant results between treatments were observed after two-way ANOVA, and no effect of rank was found for the monoamines and their metabolites (Table 4). The levels of 5-HT were significantly lower in TS treatment, 5HIAA levels were non-significant between treatments, and the serotonergic activity ratio (5HIAA/5-HT) was significantly higher in TS treatment (Fig. 4A–C). Dopaminergic activity was non-significant between treatments (Fig. 4D–F).

**Table 4:** Results of two-way ANOVA between treatments (with or without TS) and ranks (winner and loser fish) after dyadic contest for monoamines and their metabolites, as well as cortisol. *N* = 10 pairs for each treatment (5 winners and 5 losers)

	two-way ANOVA results					
Variables	With TS vs Without TS		Winners vs Losers		Interaction	
	F_***(1,16)***_	*P* value	F_***(1,16)***_	*P* value	F_***(1,16)***_	*P* value
5-HT	6.88	**0.02**	0.09	0.77	1.86	0.19
5HIAA	0.01	0.92	0.04	0.85	0.41	0.53
5HIAA/5-HT	6.06	**0.03**	0.42	0.52	0.11	0.75
DA	0.62	0.44	0.02	0.88	1.16	0.30
DOPAC	0.06	0.80	0.03	0.87	0.91	0.35
DOPAC/DA	1.61	0.22	0.01	0.91	0.01	0.91
Cortisol	2.14	**0.003**	0.11	0.74	1.18	0.29

### Cortisol

**Fig. 5:**
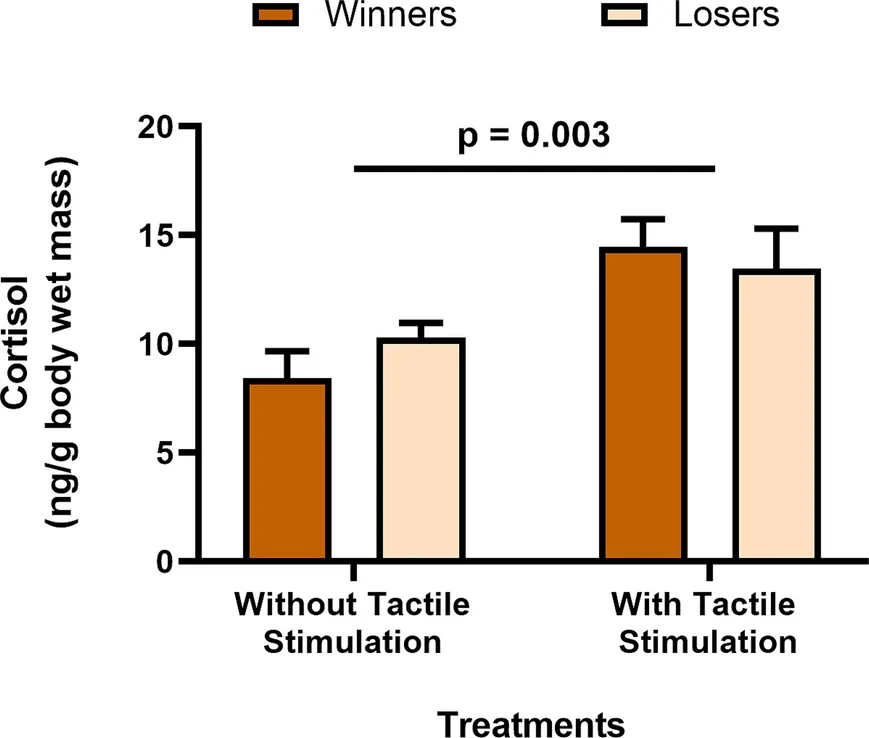
The whole-body cortisol levels. Values are shown for winners and loser fish. *P*-value between bars compares between treatments after two-way ANOVA (*N* = 10 pairs, being 5 winners and 5 losers *per* treatment). There was no effect of rank. Data are mean ± S.E

Whole-body cortisol levels were significantly higher in TS treatment and showed no effect of rank (Table 4; Fig. 5).

### Correlations between aggressive behavior and physiological variables

Some correlations were found between the aggressive variables analyzed in the two treatments (Table 5). All behavioral units of the mirror test refer to the test that occurred after the treatments, since the fights and the collection of biological material also occurred after the stimulation period. After the Holm-Bonferroni adjustment, only three correlations remained significant. In TS treatment in the second mirror test, attacks was positively correlated with cortisol (adjusted *p* = 0.034). In the treatment without TS, in dyadic fights, we found significant correlations in attacks vs DOPAC/DA (adjusted *p* = 0.043) and latency to initiate attacks vs DOPAC (adjusted *p* = 0.024). Crossings were tested only once; then, the results remained the same, as reported in Table 5.

**Table 5:**
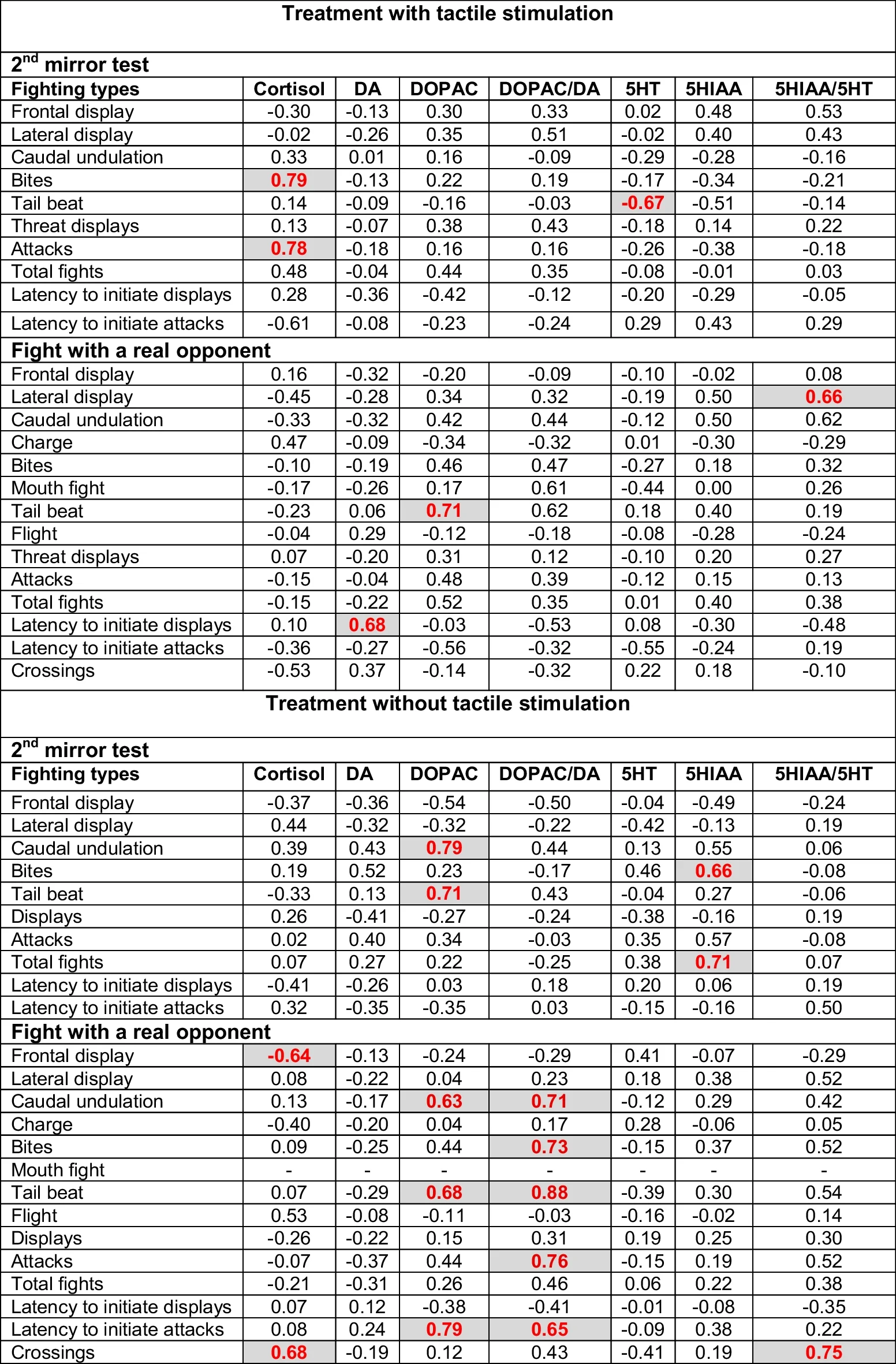
Results of the Spearman’s rank correlation test between behavioral variables and physiological ones after the stimulation period, in treatments with and without tactile stimulation (*N* = 10 for each treatment). Significant correlations (*p* ≤ 0.05) are highlighted in red and gray.

### Growth

No differences were found between treatments in any growth-related variable (Table 6). However, condition factor, although similar between treatments at the beginning of the experiments, increased significantly less in TS than in the control treatment (mixed model ANOVA; between treatment (F_(1,38)_ = 8.06, *p* = 0.007)), but both treatments showed an increment in K (within treatments F_(1,38)_ = 55.56, *p* = 0.000001). Tukey’s post hoc comparison is in Table 6.

**Table 6:** Biometry data and growth indicators in the fish that received or did not receive TS (*N* = 20 *per* treatment). Standard length and weight were taken on the first day and the 25th. Data are mean ± SE and were analyzed by unpaired t-test. Condition factor (K) was compared by Tukey’s post hoc test after mixed model ANOVA

Biometric variables	Treatments		*t*-value	*p****-***value
	Without tactile stimulation	With tactile stimulation		
Initial standard length (cm)	3.42 ± 0.05	3.44 ± 0.05	− 0.26	0.79
Final standard length (cm)	3.47 ± 0.05	3.53 ± 0.05	− 0.83	0.41
Standard length gain (cm)	0.05 ± 0.05	0.09 ± 0.02	− 0.80	0.43
Initial weight (g)	1.31 ± 0.08	1.23 ± 0.05	0.86	0.39
Final weight (g)	1.77 ± 0.11	1.60 ± 0.06	1.38	0.20
Weight gain (g)	0.46 ± 0.07	0.37 ± 0.04	1.12	0.27
Specific growth rate (%)	1.23 ± 0.16	1.07 ± 0.11	0.83	0.41
			***p*-value after Tukey’s test between treatments**	
Initial K	3.23 ± 0.128	2.99 ± 0.08	0.55	
Final K	4.21 ± 0.17	3.64 ± 0.13	**0.01**	
***p*-value after Tukey’s test within treatments**	**0.0002**	**0.001**		

## Discussion

In this study, we tested the hypothesis that TS improves welfare by reducing aggression in *B. splendens*. We observed that the fish spontaneously crossed the apparatus with TS throughout the test period, demonstrating the apparatus’s adequacy. However, individuals previously exposed to TS treatment did not exhibit decreased aggressiveness but instead appeared more reactive during fighting, both behaviorally and physiologically (in cortisol and 5-HT activity ratios). However, in the case of sustained activation of 5-HT turnover, stress levels and a lower K increment could also mean that enrichment with TS is stressful for this species. Thus, instead of acting as an overall positive sensory enrichment, TS may have been perceived as an unpredictable mechanical stimulus. In territorial species, such as *B. splendens*, this stimulus may be interpreted as an environmental disturbance rather than affiliative contact.

The tactile apparatus did not prevent fish from exploring the environment as they crossed through it constantly from the first day. The lower number of crossings in the apparatus with bristles was expected, since silicone bristles can function as a mechanical barrier, even though they are soft, as also occurred with Nile tilapia (Bolognesi et al. 2019; Gauy et al. 2021) and gilthead seabream (Soares et al. 2026). Therefore, the TS device was suitable for testing the hypothesis in Betta fish. Unlike Nile tilapia, Betta fish did not require 3 to 4 days to adjust to the apparatus, probably because they explore their environment more, even when fish are isolated (Ramos and Gonçalves 2019).

The mirror test is used to assess individual aggressiveness in several species of fish, which usually do not identify their own image (Barreto et al. 2009), including *B. splendens* (Ramos et al. 2021; Oliveira et al. 2022). Although not natural and subject to some criticism (Balzarini et al. 2014), the advantage of using the mirror test lies in the potential control of the opponent’s behavior, with results attributed only to the behavior of the focal individual (Reddon et al. 2012). In this test, however, we found no significant differences in latency or frequency of specific behaviors, indicating similar motivation to initiate and escalate the fight. As observed by Silva et al. (2025), there is a high individual variation in aggressive behavior against a mirror in *B. splendens* and a habituation to the mirror image. High variability can sometimes reduce the probability of significant differences when comparing groups by ANOVA. However, we found no evidence of habituation to the mirror, as the results before and after the TS experiments were similar. From this data, we can conclude that there was no effect of TS on individual aggressive motivation.

In contrast to the mirror test, in a fight against a real opponent, an individual’s behavior depends on the other’s actions (Reddon et al. 2012). Thus, we paired individuals from the same treatment to evaluate the effects of TS on the mutual motivation to fight. Again, we observed no difference between the treatments with and without TS in the latency to initiate threats and attacks. Nevertheless, fish that received TS showed a higher frequency of lateral and frontal display than the control fish. Those are the main behaviors indicating territorial defense against conspecifics in *B. splendens* (Forsatkar et al. 2017). Although frontal displays and lateral displays were higher in TS treatment, this was not confirmed when summing the four threat behavioral units. In fact, depending on the fish species and behavior, one behavioral variable can better represent the aggressive context. For example, in some cichlids, lateral threat alone represents the whole repertoire of threats (Noleto-Filho et al. 2019). Therefore, frontal and lateral display in *B. splendens* indicates a high motivation to fight after TS, even though the remaining behaviors are similar. We also found significant differences between winners and losers in attacks, threat displays, and flights, confirming that a social hierarchy was established during the 20 min of video recordings. Those variables, however, were not affected by the treatment with or without TS. Again, this reinforces the importance of testing a set of behavioral variables to better understand aggressive interactions in different fish species (Noleto-Filho et al. 2019).

Tactile perception requires machinery to translate sensory stimuli into a message of social touch. Such machinery is, nevertheless, understudied in fish, although tactile receptors other than those from lateral lines are found in fish’s body (Devitsina and Lapshin 2020). On the other hand, a perception of a negative-valence sense also requires specific physiological machinery. Therefore, it is plausible to assume that bettas have the physiological machinery to perceive TS as body touch, even though it does not produce soothing effects as found in other species.

Species that naturally experience frequent tactile contact during social interactions may interpret TS as affiliative or rewarding. For instance, TS reduces aggression in Nile tilapia (Gauy et al. 2022), which, despite being a territorial species, has a rich repertoire of social behavior (Gonçalves-de-Freitas et al. 2019), thereby causing contact that provides natural TS, particularly during courtship (Fessehaye et al. 2006*).* The same is observed for white seabream (*Diplodus sargus*), whose social behavior is associated with a preference for TS (Gauy et al. 2026). In contrast, solitary and highly territorial species may perceive similar stimuli as intrusive or threatening. Betta fish, for example, usually live in isolation and are highly territorial (Iwata et al. 2021). In addition, males are reared in isolation from the beginning of their lives in fish farms. Therefore, prolonged social isolation could be one of the reasons for the lack of effects of TS on aggressive behavior, as males of *B. splendens* are usually deprived of physical contact with conspecifics. Another possible explanation is the intense and prolonged artificial selection that the fish underwent, selecting individuals for extreme aggression (Verbeek et al. 2007; Iwata et al. 2021). This species has a long history of artificial selection, being cultivated for aggression to be used in clandestine fights 600 years ago (Sermwatanakul 2019). This long-term selection resulting from breeding different lineages has selected highly aggressive males and females (Verbeek et al. 2007; Ramos & Gonçalves 2019). Thus, the genetic characteristics selected for aggressiveness can be so intense that they prevent the reaction to soothing stimuli such as TS.

Growth parameters were similar between treatments, despite the differences in some behavioral and physiological variables. Broadly speaking, growth rate is the net balance between energy intake and energy allocation (Kooijman 2010). Fish subject to TS or control were fed the same amount of food; therefore, energy intake is supposed to be similar between treatments. However, we found no effect of TS on growth parameters, despite the fish in the TS group crossing the center of the aquarium less frequently and therefore expending less energy. Conversely, TS fish also showed higher stress levels, an important source of energy depletion (Sadoul and Vijayan 2016), therefore balancing the SGR and making it similar between treatments. Despite the non-significant differences in growth parameters between treatments, the K increased less in TS-treated fish. K is the ratio of body mass and the cube of length and is considered an indirect metric of a fish’s health and energy, operating under the assumption that heavier fish of a given length possess greater energy reserves (Bolger and Connolly 1989; Mozsár et al. 2015). Here, fish from control showed a slightly higher percentage of weight increment than TS (35% vs 30%, respectively) while showing a lower length increment percentage (15% vs 26%), indicating that the energy reserves in TS were used to increase length instead of weight.

Despite these results, we can conclude that the animals in both treatments did not enter the distress phase, as there was no negative growth or K in either TS or control fish (Sopinka et al. 2016). The cortisol levels observed in this study likely reflect an acute response to aggressive interactions rather than long-term metabolic shifts, as indicated by SGR data. This is consistent with findings by Ramos et al. (2021), who documented a rise in cortisol following contest in *B. splendens*. Generally, competitive or conflicting environments trigger cortisol release as part of the primary stress response (Wendelaar Bonga 1997; Øverli et al. 2005). While chronic elevation can suppress aggression (Summers et al. 2005), acute spikes may enhance aggressive displays and the motivation to defend territory (Summers and Winberg 2006). Although cortisol alone does not explain the whole mechanism of aggressive behavior (Serra et al. 2015), the acute increase measured immediately post-interaction likely explains the heightened motivation observed in fish subjected to TS. This assertion is partially supported by positive correlations between overt fights and cortisol in the mirror.

Tactile stimulation (TS) applied to the fish body does not always reduce cortisol levels, even when it decreases aggressive interactions. For example, Nile tilapia exposed to TS exhibit a marked reduction in aggression without a corresponding decrease in cortisol concentrations (Bolognesi et al. 2019; Gauy et al. 2022). In contrast, TS attenuates the cortisol response to a non-social stressor (confinement) in the surgeonfish *Ctenochaetus striatus* (Soares et al., 2011) and the gilthead seabream *Sparus aurata* (Soares et al. 2026), compared with fish that were not stressed. In the present study, however, we did not include a non-stressed control group, which prevents us from determining the effect of TS alone on stress levels in socially challenged fish. Although this limitation prevents a complete understanding of the effects of TS on the welfare of *B. splendens*, our findings provide the first evidence that this type of sensory enrichment may increase, rather than reduce, the stress response in fish.

DA is a crucial neurotransmitter that affects movement, emotions, and the brain’s reward system (Marsden 2006). In fish, this monoamine plays fundamental roles in regulating behavior. This neurotransmitter helps control vigilance and alertness, including anticipatory responses to stimuli associated with a reward (Heimovics et al. 2009). TS is perceived as a reward for several vertebrates; therefore, DA levels are expected to increase under a positively perceived stimulus. For instance, TS of rats’ skin increases DA release in the nucleus accumbens (Maruyama et al. 2012). In the mutualistic interaction between client-cleaner fish, the client fish shows higher levels of DA in the forebrain when receiving TS from the cleaner fish (Abreu et al. 2018). In our study, however, DA levels remained unchanged, indicating no activation of the neural reward pathway and, consequently, showing no positive effect from TS in this species. On the other hand, we found strong positive correlations between DA metabolites and aggressive behavior in the control treatment. This positive correlation probably reflects decision-making; i.e., animals with higher brain DA could be more motivated to fight (Winberg and Nilsson 1993; Antunes et al. 2022).

5-HT is a neurotransmitter known for its influence on fish behavior, particularly aggression (Winberg and Thörnqvist 2016) and the stress response (Yuan et al. 2025; Staven et al. 2026). Generally, less aggressive individuals exhibit higher serotonergic activity, whereas low 5-HT levels are associated with increased aggression and boldness (Lillesaar 2011; Winberg and Thörnqvist 2016). This inhibitory effect is evident in *B. splendens*, where both 5-HT administration and inhibition of its reuptake by fluoxetine reduce aggressive displays (Clotfelter et al. 2007; Greene and Szalda-Petree 2022). Conversely, social stress and isolation typically deplete 5-HT while increasing its metabolite, 5-HIAA, though these effects vary by species and coping style (Shams et al. 2017; Lim et al. 2023; Yuan et al. 2025). Our findings are consistent with these mechanisms, as fish subjected to TS exhibited higher cortisol levels, lower 5-HT, and higher 5-HIAA/5-HT ratio than control individuals. Nevertheless, some correlations that would provide additional explanation regarding serotonergic activity and aggressive behavior became non-significant after Holm-Bonferroni adjustment. The absence of significant correlations should be interpreted with caution because the statistical power of correlation tests is strongly dependent on sample size. With small sample sizes, only relatively strong associations are likely to reach statistical significance (Zar 2010), which could have happened in our study, as we tested only 10 replicates each physiological variable. Finally, cortisol and 5-HT turnover were positively correlated with crossings in the control. However, we need to remember that, in this condition, the fish just crossed over the aquarium without getting any tactile stimulus. This would mean that displacement per se can be stressful for the fish, although less than when receiving physical stimulus.

The mechanisms underlying TS in fish are still poorly understood. However, this study demonstrates that body TS does not reduce aggressive behavior in *B. splendens*. Conversely, this type of enrichment increases stress and heightens motivation to fight. By looking at the whole welfare indicators, particularly cortisol and 5-HT turnover, we conclude that this type of enrichment is not positive for *B. splendens*, therefore highlighting that environmental enrichment strategies cannot be universally applied across species, as their perception and effectiveness depend on the ecological characteristics and behavioral repertoire of each species. Although TS brings a positive effect to other fish species, it is not efficient to improve *B. splendens*’ welfare.

## Supplementary Information

Below is the link to the electronic supplementary material.

ESM 1 (DOCX 140 KB)

## Data Availability

Data used in this research is attached as a supplementary material—spreadsheet.
